# Methyl 2-(1,1,3-trioxo-2,3-dihydro-1,2-benzothia­zol-2-yl)acetate: a monoclinic polymorph

**DOI:** 10.1107/S1600536810012006

**Published:** 2010-04-10

**Authors:** Muhammad Zia-ur-Rehman, Muhammad Nadeem Arshad, Shafaq Mubarak, Islam Ullah Khan

**Affiliations:** a Applied Chemistry Research Centre, PCSIR Laboratories Complex, Lahore 54600, Pakistan; b Department of Chemistry, Government College University, Lahore 54000, Pakistan

## Abstract

In the title compound, C_10_H_9_NO_5_S, the fused ring system and the planar (r.m.s. deviation = 0.0037 Å) methoxy­carbonyl­methyl side chain form a dihedral angle of 84.67 (10)°. The crystal structure is stabilized by inter­molecular C—H⋯O hydrogen bonds. A triclinic polymorph of the title compound is already known [Siddiqui *et al.* (2008 ▶). *Acta Cryst*. E**64**, o859].

## Related literature

For the synthesis and biological activity of related compounds, see: Ahmad *et al.* (2010 ▶); Zia-ur-Rehman *et al.* (2005 ▶, 2006 ▶, 2007 ▶). For a related structure, see: Arshad *et al.* (2009 ▶). For the triclinic polymorph, see: Siddiqui *et al.* (2008 ▶).
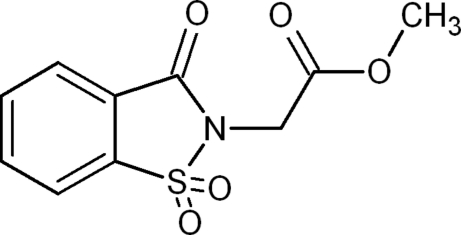


## Experimental

### 

#### Crystal data

C_10_H_9_NO_5_S
*M* *_r_* = 255.24
Monoclinic, 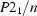

*a* = 8.9418 (4) Å
*b* = 12.7595 (6) Å
*c* = 10.3145 (5) Å
β = 107.300 (1)°
*V* = 1123.57 (9) Å^3^
*Z* = 4
Mo *K*α radiation
μ = 0.30 mm^−1^
*T* = 296 K
0.43 × 0.41 × 0.13 mm

#### Data collection

Bruker APEXII CCD area-detector diffractometer
Absorption correction: multi-scan (*SADABS*; Sheldrick, 2004 ▶) *T*_min_ = 0.883, *T*_max_ = 0.962
12621 measured reflections
2796 independent reflections
2022 reflections with *I* > 2σ(*I*)
*R*_int_ = 0.025

#### Refinement

*R*[*F* ^2^ > 2σ(*F* ^2^)] = 0.039
*wR*(*F* ^2^) = 0.115
*S* = 1.03
2796 reflections
155 parameters
H-atom parameters constrained
Δρ_max_ = 0.24 e Å^−3^
Δρ_min_ = −0.32 e Å^−3^

### 

Data collection: *APEX2* (Bruker, 2007 ▶); cell refinement: *SAINT* (Bruker, 2007 ▶); data reduction: *SAINT*; program(s) used to solve structure: *SHELXS97* (Sheldrick, 2008 ▶); program(s) used to refine structure: *SHELXL97* (Sheldrick, 2008 ▶); molecular graphics: *PLATON* (Spek, 2009 ▶); software used to prepare material for publication: *WinGX* (Farrugia, 1999 ▶) and *PLATON*.

## Supplementary Material

Crystal structure: contains datablocks I, global. DOI: 10.1107/S1600536810012006/bt5235sup1.cif

Structure factors: contains datablocks I. DOI: 10.1107/S1600536810012006/bt5235Isup2.hkl

Additional supplementary materials:  crystallographic information; 3D view; checkCIF report

## Figures and Tables

**Table 1:** Hydrogen-bond geometry (Å, °)

*D*—H⋯*A*	*D*—H	H⋯*A*	*D*⋯*A*	*D*—H⋯*A*
C8—H8*B*⋯O1^i^	0.97	2.46	3.371 (3)	156
C2—H2⋯O4^ii^	0.93	2.59	3.455 (3)	155
C3—H3⋯O2^ii^	0.93	2.50	3.331 (3)	148
C10—H10*C*⋯O2^iii^	0.96	2.52	3.419 (3)	156

Symmetry codes: (i) 
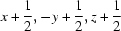
; (ii) 
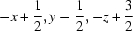
; (iii) 
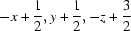
.

**Fig. 1.**
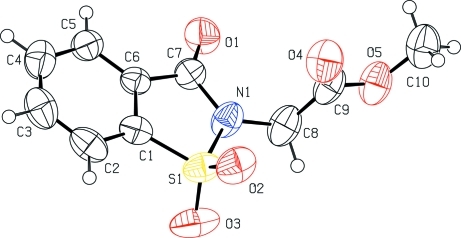
The title molecule with the displacement ellipsoids plotted at 50% probability level.

## supplementary crystallographic information

### Comment

A triclinic polymorph (Siddiqui *et al.*, 2008) of the title compound, methyl (1,1-dioxido-3-oxo-1,2-benzisothiazol-2(3*H*)-yl)acetate (I), has already been reported. In continuation of our work on the synthesis (Zia-ur-Rehman *et al.*, 2006; Ahmad *et al.*, 2010), and crystal structures (Zia-ur-Rehman *et al.*, 2007; Arshad *et al.*, 2009) of various 1,2-benzothiazine-1,1-dioxides, we have been able to crystallize a monoclinic polymorph of the title compound. The methoxycarbonylmethyl side chain is oriented at a dihedral angle of 84.67 (10)° with respect to the fused ring system. The molecules are connected by intermolecular C–H···O interactions giving rise to a three dimentional network.

### Experimental

The title compound   was prepared following the procedure  reported earlier (Zia-ur-Rehman *et al.*, 2005). Crystals suitable for X-ray crystallography were grown in chloroform by slow evaporation at 313 K.

### Refinement

H-atoms were included in the refinement  at geometrically idealized positions with aryl, methylene and methyl C—H distances 0.95, 0.99 and 0.98 Å, respectively, and U(H) = 1.2 times U_eq_(C).

### Crystal data

**Table:**

C_10_H_9_NO_5_S	*F*(000) = 528
*M**_r_* = 255.24	*D*_x_ = 1.509 Mg m^−^^3^
Monoclinic, *P*2_1_/*n*	Mo *K*α radiation, λ = 0.71073 Å
Hall symbol: -P 2yn	Cell parameters from 4707 reflections
*a* = 8.9418 (4) Å	θ = 2.7–26.7°
*b* = 12.7595 (6) Å	µ = 0.30 mm^−^^1^
*c* = 10.3145 (5) Å	*T* = 296 K
β = 107.300 (1)°	Needles, colourless
*V* = 1123.57 (9) Å^3^	0.43 × 0.41 × 0.13 mm
*Z* = 4	

### Data collection

**Table:**

Bruker APEXII CCD area-detector diffractometer	2796 independent reflections
Radiation source: fine-focus sealed tube	2022 reflections with *I* > 2σ(*I*)
graphite	*R*_int_ = 0.025
phi and ω scans	θ_max_ = 28.4°, θ_min_ = 2.6°
Absorption correction: multi-scan (*SADABS*; Sheldrick, 2004)	*h* = −11→11
*T*_min_ = 0.883, *T*_max_ = 0.962	*k* = −16→17
12621 measured reflections	*l* = −13→12

### Refinement

**Table:**

Refinement on *F*^2^	Primary atom site location: structure-invariant direct methods
Least-squares matrix: full	Secondary atom site location: difference Fourier map
*R*[*F*^2^ > 2σ(*F*^2^)] = 0.039	Hydrogen site location: inferred from neighbouring sites
*wR*(*F*^2^) = 0.115	H-atom parameters constrained
*S* = 1.02	*w* = 1/[σ^2^(*F*_o_^2^) + (0.0523*P*)^2^ + 0.2836*P*] where *P* = (*F*_o_^2^ + 2*F*_c_^2^)/3
2796 reflections	(Δ/σ)_max_ < 0.001
155 parameters	Δρ_max_ = 0.24 e Å^−^^3^
0 restraints	Δρ_min_ = −0.32 e Å^−^^3^

### Special details

**Table:**

Geometry. All s.u.'s (except the s.u. in the dihedral angle between two l.s. planes) are estimated using the full covariance matrix. The cell s.u.'s are taken into account individually in the estimation of s.u.'s in distances, angles and torsion angles; correlations between s.u.'s in cell parameters are only used when they are defined by crystal symmetry. An approximate (isotropic) treatment of cell s.u.'s is used for estimating s.u.'s involving l.s. planes.
Refinement. Refinement of *F*^2^ against ALL reflections. The weighted *R*-factor wR and goodness of fit *S* are based on *F*^2^, conventional *R*-factors *R* are based on *F*, with *F* set to zero for negative *F*^2^. The threshold expression of *F*^2^ > 2σ(*F*^2^) is used only for calculating *R*-factors(gt) etc. and is not relevant to the choice of reflections for refinement. *R*-factors based on *F*^2^ are statistically about twice as large as those based on *F*, and *R*- factors based on ALL data will be even larger.

### Fractional atomic coordinates and isotropic or equivalent isotropic displacement parameters (Å^2^)

**Table:**

	*x*	*y*	*z*	*U*_iso_*/*U*_eq_	
S1	0.38220 (5)	0.08616 (4)	0.63435 (4)	0.05841 (18)	
O1	0.16399 (18)	0.16167 (11)	0.27984 (13)	0.0700 (4)	
O2	0.33381 (18)	0.13244 (13)	0.74048 (13)	0.0744 (4)	
O3	0.54303 (16)	0.05974 (16)	0.66339 (16)	0.0892 (5)	
O4	0.17124 (19)	0.34008 (12)	0.54605 (16)	0.0776 (4)	
O5	0.33001 (19)	0.44091 (12)	0.46863 (15)	0.0729 (4)	
N1	0.3269 (2)	0.16078 (13)	0.49608 (15)	0.0602 (4)	
C1	0.25915 (19)	−0.01610 (14)	0.55496 (17)	0.0491 (4)	
C2	0.2399 (3)	−0.11272 (17)	0.6095 (2)	0.0649 (5)	
H2	0.2951	−0.1303	0.6984	0.078*	
C3	0.1358 (3)	−0.18129 (17)	0.5268 (2)	0.0720 (6)	
H3	0.1196	−0.2466	0.5605	0.086*	
C4	0.0549 (3)	−0.15539 (16)	0.3951 (2)	0.0681 (6)	
H4	−0.0144	−0.2036	0.3413	0.082*	
C5	0.0746 (2)	−0.05929 (15)	0.3415 (2)	0.0556 (4)	
H5	0.0201	−0.0423	0.2523	0.067*	
C6	0.17697 (18)	0.01082 (13)	0.42324 (16)	0.0449 (4)	
C7	0.2156 (2)	0.11722 (15)	0.38655 (17)	0.0508 (4)	
C8	0.3914 (3)	0.26501 (17)	0.4912 (2)	0.0697 (6)	
H8A	0.4143	0.2735	0.4056	0.084*	
H8B	0.4892	0.2714	0.5636	0.084*	
C9	0.2822 (2)	0.35100 (16)	0.50537 (18)	0.0603 (5)	
C10	0.2369 (3)	0.53266 (18)	0.4757 (2)	0.0805 (7)	
H10A	0.1314	0.5223	0.4189	0.121*	
H10B	0.2809	0.5930	0.4450	0.121*	
H10C	0.2368	0.5433	0.5678	0.121*	

### Atomic displacement parameters (Å^2^)

**Table:**

	*U*^11^	*U*^22^	*U*^33^	*U*^12^	*U*^13^	*U*^23^
S1	0.0530 (3)	0.0830 (4)	0.0369 (2)	0.0002 (2)	0.00972 (18)	−0.0025 (2)
O1	0.0958 (11)	0.0643 (9)	0.0439 (7)	0.0047 (7)	0.0117 (7)	0.0109 (6)
O2	0.0837 (10)	0.0975 (11)	0.0418 (7)	−0.0028 (8)	0.0183 (7)	−0.0136 (7)
O3	0.0478 (8)	0.1463 (16)	0.0655 (10)	0.0058 (9)	0.0046 (7)	−0.0070 (10)
O4	0.0842 (10)	0.0759 (10)	0.0847 (11)	−0.0175 (8)	0.0436 (9)	0.0019 (8)
O5	0.0924 (11)	0.0655 (9)	0.0701 (9)	−0.0261 (8)	0.0383 (8)	−0.0062 (7)
N1	0.0750 (10)	0.0620 (10)	0.0416 (8)	−0.0159 (8)	0.0140 (7)	−0.0033 (7)
C1	0.0490 (9)	0.0595 (10)	0.0414 (8)	0.0098 (8)	0.0176 (7)	0.0036 (7)
C2	0.0748 (13)	0.0683 (13)	0.0589 (12)	0.0215 (10)	0.0312 (10)	0.0200 (10)
C3	0.0869 (15)	0.0536 (12)	0.0892 (16)	0.0044 (11)	0.0471 (13)	0.0101 (11)
C4	0.0695 (13)	0.0585 (12)	0.0837 (15)	−0.0092 (10)	0.0340 (11)	−0.0112 (11)
C5	0.0519 (10)	0.0594 (11)	0.0543 (10)	0.0007 (8)	0.0139 (8)	−0.0039 (8)
C6	0.0437 (8)	0.0503 (9)	0.0416 (8)	0.0061 (7)	0.0140 (7)	0.0016 (7)
C7	0.0589 (10)	0.0548 (10)	0.0387 (9)	0.0022 (8)	0.0146 (8)	−0.0011 (7)
C8	0.0799 (14)	0.0725 (14)	0.0637 (12)	−0.0248 (11)	0.0319 (11)	−0.0117 (10)
C9	0.0734 (13)	0.0662 (12)	0.0434 (10)	−0.0248 (10)	0.0203 (9)	−0.0088 (8)
C10	0.1110 (19)	0.0673 (14)	0.0676 (14)	−0.0142 (13)	0.0335 (13)	−0.0033 (11)

### Geometric parameters (Å, °)

**Table:**

S1—O3	1.4196 (15)	C3—C4	1.376 (3)
S1—O2	1.4199 (14)	C3—H3	0.9300
S1—N1	1.6630 (16)	C4—C5	1.378 (3)
S1—C1	1.7457 (19)	C4—H4	0.9300
O1—C7	1.202 (2)	C5—C6	1.375 (2)
O4—C9	1.194 (2)	C5—H5	0.9300
O5—C9	1.319 (2)	C6—C7	1.478 (3)
O5—C10	1.451 (3)	C8—C9	1.505 (3)
N1—C7	1.381 (2)	C8—H8A	0.9700
N1—C8	1.456 (3)	C8—H8B	0.9700
C1—C6	1.382 (2)	C10—H10A	0.9600
C1—C2	1.387 (3)	C10—H10B	0.9600
C2—C3	1.374 (3)	C10—H10C	0.9600
C2—H2	0.9300		
			
O3—S1—O2	117.25 (9)	C6—C5—H5	120.8
O3—S1—N1	109.94 (10)	C4—C5—H5	120.8
O2—S1—N1	110.00 (10)	C5—C6—C1	120.23 (17)
O3—S1—C1	112.28 (10)	C5—C6—C7	127.21 (16)
O2—S1—C1	112.24 (9)	C1—C6—C7	112.55 (15)
N1—S1—C1	92.32 (8)	O1—C7—N1	123.13 (17)
C9—O5—C10	116.58 (17)	O1—C7—C6	127.78 (17)
C7—N1—C8	122.39 (16)	N1—C7—C6	109.08 (15)
C7—N1—S1	115.42 (13)	N1—C8—C9	112.79 (16)
C8—N1—S1	122.19 (14)	N1—C8—H8A	109.0
C6—C1—C2	121.73 (18)	C9—C8—H8A	109.0
C6—C1—S1	110.62 (13)	N1—C8—H8B	109.0
C2—C1—S1	127.65 (15)	C9—C8—H8B	109.0
C3—C2—C1	117.17 (19)	H8A—C8—H8B	107.8
C3—C2—H2	121.4	O4—C9—O5	125.2 (2)
C1—C2—H2	121.4	O4—C9—C8	125.44 (19)
C2—C3—C4	121.4 (2)	O5—C9—C8	109.32 (17)
C2—C3—H3	119.3	O5—C10—H10A	109.5
C4—C3—H3	119.3	O5—C10—H10B	109.5
C3—C4—C5	121.2 (2)	H10A—C10—H10B	109.5
C3—C4—H4	119.4	O5—C10—H10C	109.5
C5—C4—H4	119.4	H10A—C10—H10C	109.5
C6—C5—C4	118.33 (19)	H10B—C10—H10C	109.5
			
O3—S1—N1—C7	115.77 (16)	C2—C1—C6—C5	−1.0 (3)
O2—S1—N1—C7	−113.66 (15)	S1—C1—C6—C5	179.06 (13)
C1—S1—N1—C7	1.03 (15)	C2—C1—C6—C7	179.97 (16)
O3—S1—N1—C8	−64.37 (18)	S1—C1—C6—C7	0.06 (18)
O2—S1—N1—C8	66.20 (18)	C8—N1—C7—O1	0.0 (3)
C1—S1—N1—C8	−179.10 (16)	S1—N1—C7—O1	179.84 (15)
O3—S1—C1—C6	−113.27 (13)	C8—N1—C7—C6	179.00 (16)
O2—S1—C1—C6	112.14 (13)	S1—N1—C7—C6	−1.14 (19)
N1—S1—C1—C6	−0.60 (13)	C5—C6—C7—O1	0.7 (3)
O3—S1—C1—C2	66.84 (19)	C1—C6—C7—O1	179.61 (18)
O2—S1—C1—C2	−67.76 (18)	C5—C6—C7—N1	−178.26 (17)
N1—S1—C1—C2	179.51 (17)	C1—C6—C7—N1	0.6 (2)
C6—C1—C2—C3	0.3 (3)	C7—N1—C8—C9	77.7 (2)
S1—C1—C2—C3	−179.82 (14)	S1—N1—C8—C9	−102.1 (2)
C1—C2—C3—C4	0.4 (3)	C10—O5—C9—O4	−1.6 (3)
C2—C3—C4—C5	−0.4 (3)	C10—O5—C9—C8	179.77 (17)
C3—C4—C5—C6	−0.4 (3)	N1—C8—C9—O4	16.0 (3)
C4—C5—C6—C1	1.0 (3)	N1—C8—C9—O5	−165.36 (16)
C4—C5—C6—C7	179.88 (17)		

### Hydrogen-bond geometry (Å, °)

**Table:**

*D*—H···*A*	*D*—H	H···*A*	*D*···*A*	*D*—H···*A*
C8—H8B···O1^i^	0.97	2.46	3.371 (3)	156
C2—H2···O4^ii^	0.93	2.59	3.455 (3)	155
C3—H3···O2^ii^	0.93	2.50	3.331 (3)	148
C10—H10C···O2^iii^	0.96	2.52	3.419 (3)	156

Symmetry codes: (i) *x*+1/2, −*y*+1/2, *z*+1/2; (ii) −*x*+1/2, *y*−1/2, −*z*+3/2; (iii) −*x*+1/2, *y*+1/2, −*z*+3/2.
